# Accelerated biodegradation of silk sutures through matrix metalloproteinase activation by incorporating 4-hexylresorcinol

**DOI:** 10.1038/srep42441

**Published:** 2017-02-13

**Authors:** You-Young Jo, HaeYong Kweon, Dae-Won Kim, Min-Keun Kim, Seong-Gon Kim, Jwa-Young Kim, Weon-Sik Chae, Sam-Pyo Hong, Young-Hwan Park, Si Young Lee, Je-Yong Choi

**Affiliations:** 1 Rural Development Administration, Wanju-Gun 55365, South Korea; 2 Dept. of Oral Biochemistry, College of Dentistry, Gangneung-Wonju National University, Gangneung 25457, South Korea; 3 Dept. of Oral and Maxillofacial Surgery, College of Dentistry, Gangneung-Wonju National University, Gangneung 25457, South Korea; 4 Dept. of Oral and Maxillofacial Surgery, College of Medicine, Hallym University, Anyang 14068, South Korea; 5 Analysis Research Division, Daegu Center, Korea Basic Science Institute, South Korea; 6 Dept. of Oral Pathology, College of Dentistry, Seoul National University, Seoul 03080, South Korea; 7 Department of Biosystems & Biomaterials Science and Engineering, Seoul National University, South Korea; 8 Dept. of Oral Microbiology, College of Dentistry, Gangneung-Wonju National University, Gangneung 25457, South Korea; 9 School of Biochemistry and Cell Biology, Skeletal Diseases Genome Research Center, Kyungpook National University, Daegu 700-842, Korea

## Abstract

Silk suture material is primarily composed of silk fibroin and regarded as a non-resorbable material. It is slowly degraded by proteolysis when it is implanted into the body. 4-Hexylresorcinol (4HR) is a well-known antiseptic. In this study, the biodegradability of 4HR-incorporated silk sutures were compared to that of untreated silk sutures and polyglactin 910 sutures, a commercially available resorbable suture. 4HR-incorporated silk sutures exhibited anti-microbial properties. Matrix metalloproteinase (MMP) can digest a wide spectrum of proteins. 4HR increased MMP-2, -3, and -9 expression in RAW264.7 cells. MMP-2, -3, and -9 were able to digest not only silk fibroin but also silk sutures. Consequently, 59.5% of the 4HR-incorporated silk suture material remained at 11 weeks after grafting, which was similar to that of polyglactin 910 degradation (56.4% remained). The residual amount of bare silk suture material at 11 weeks after grafting was 91.5%. The expression levels of MMP-2, -3 and -9 were high in the 4HR-incorporated silk suture-implanted site 12 weeks after implantation. In conclusion, 4HR-treated silk sutures exhibited anti-microbial properties and a similar level of bio-degradation to polyglactin 910 sutures and induced higher expression of MMP-2, -3, and -9 in macrophages.

Suture materials are some of the most widely used biomaterials in the surgical fields. The gross scale of the suture market has increased by several million dollars annually^1^,^2^. The purpose of suturing is to help the natural healing process by occluding a wounded area^3^. Therefore, the ideal suture material should have both appropriate biological compatibility and physical strength. Suture materials can be classified as absorbable sutures and non-absorbable sutures^3^,^4^. When using non-biodegradable sutures, the removal of suture material is generally required. Removing sutures are clinically challenging, particularly in difficult-to-access anatomical areas or in pediatric patients. In such cases, using biodegradable sutures is recommended^5^,^6^.

Silk sutures are composed of silk fibroin protein from *Bombyx mori* (70%) and coating material (30%)^6^. Silk sutures are regarded as non-biodegradable sutures because complete bio-degradation requires approximately 2 years^6^,^7^. Because silk sutures are relatively inexpensive, they have been widely used for mucosal wound closure and vessel ligation^4^,^6^. Although silk fibroin is considered a bio-inert material^8^, many types of micro-organisms can attach to silk sutures and induce inflammation^5^. For this reason, antibiotic-incorporated silk sutures have been developed^5^. To the best of our knowledge, however, there has been no report that has discussed the anti-microbial properties of biodegradable silk sutures.

4-Hexylresorcinol (4HR) is a resorcinolic lipid and has been used as an antiseptic^9^ and food ingredient for preventing melanosis^10^. 4HR is an amphiphilic molecule because of its 2 hydroxyl groups and long alkyl group^11^. Thus, 4HR can interact with target proteins via both hydrophobic and hydrophilic interactions. Hydrophobic interactions can occur between the hydrophobic domain of the target protein and the long alkyl group of 4HR. Hydrophilic interactions can occur between the hydrophilic domain of the target protein and the 2 hydroxyl groups attached to the benzene ring. In the case of hydrophilic interactions, preferential hydration can occur depending on the type of solvent^12^. Using these 2 types of interactions, 4HR can be incorporated into proteins such as bone matrix^13^. 4HR-incorporated silk scaffolds have shown improved capabilities for use as materials for soft tissue augmentation^14^ or as membranes for guided bone regeneration^15^. Recently, we demonstrated that the 4HR-incorporated silk scaffolds showed reduced foreign body reaction and accelerated graft degradation^16^. However, the mechanism of accelerated graft degradation by 4HR-incorporation was not studied.

Many synthetic suture materials are primarily degraded by hydrolysis^17^. However, natural polymers, such as collagen, are degraded by proteolysis^18^. A group of matrix metalloproteinase (MMP) can degrade silk fibroin^19^. MMPs are enzymes that are responsible for proteolysis and are highly expressed in the acute inflammatory phase and late remodeling phase^20^. The MMPs expressed in late remodeling phases contribute to wound healing and the re-organization of connective tissue^21^. For designing smart biomaterials, an MMP-responsive drug carrier can be used for the development of a cell-responsive delivery system^22^. MMPs are mainly produced by monocytes and macrophages^23^. Whether 4HR can increase the expression of MMPs in macrophages has not been illuminated. If 4HR can increase MMP expression in macrophages, 4HR-incorporated silk sutures can be degraded based on the schedule of macrophage activation during the wound healing process. Silk fibroin has been widely studied as a potential drug carrier^24^.

Silk sutures incorporated with 4HR should have similar physical strength to that of untreated silk sutures for use in clinical applications. The first aim of this study was to compare the physical strength of 4HR-incorporated silk sutures to that of untreated silk sutures and commercially available biodegradable sutures. Although the Kaplan group demonstrated that MMP-1 and -2 can cause the proteolysis of silk, the degree of proteolysis is dependent on the processing method of silk fibroin^19^. For manufactured silk suture material, many steps for chemical treatment are required. Thus, the actual proteolysis of manufactured silk sutures by MMPs should be demonstrated. In addition, MMP induction in macrophages by 4HR should be demonstrated by *in vitro* and *in vivo* experiments. Finally, the biodegradation of 4HR-incorporated silk sutures should be demonstrated by *in vivo* experiments.

## Results

### Incorporation of 4HR into silk sutures and a comparative analysis of its physical strength

**Figure 1:**
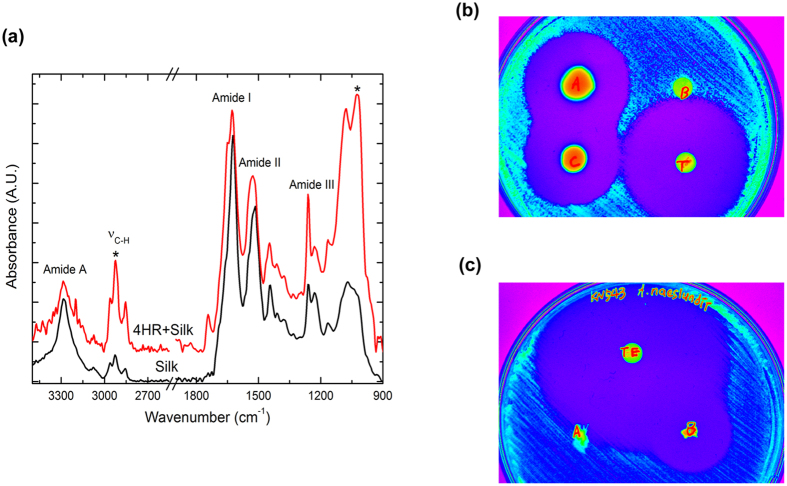
(**a**) FT-IR spectra of silk suture and 4-HR-treated silk sutures. Each amide band is indicated. For 4HR-incorporated silk, most of the amide peaks remained unchanged. However, an extra peak appeared at 1026 cm^−1^ (indicated by the asterisk) due to the C-O vibration of 4HR. The peak corresponding to the β-sheet conformation was also dramatically enhanced by 4HR treatment. (**b**) The anti-microbial assay of drug-loaded disks. The 4HR-incorporated silk disks and paper disks showed anti-bacterial properties (indicated as “A” and “C” disk, respectively). However, bare silk disks (indicated as “B” disk) did not show any inhibitory zone. The disk indicated as “T” is a tetracycline loaded disk. (**c**) The anti-microbial assay of drug-loaded sutures. Similar to the disk experiment, the 4HR-incorporated silk suture (indicated as “B” suture knot) showed anti-bacterial properties. The disk indicated as “TE” is a tetracycline loaded disk. The results are summarized in Supplementary Table S2.

Figure 1a shows the Fourier transform infrared (FT-IR) spectra of silk and 4HR-treated silk materials. Silk shows several intense vibrational absorption peaks in the mid-IR region. The absorption peak at 3282 cm^−1^ is attributed to the amide A band. The absorption peaks observed at 1624 and 1516 cm^−1^ can be assigned to the amide I and amide II bands, respectively^25^,^26^. Amide III bands appear at both 1230 and 1261 cm^−1^; the former peak is attributed to the random coil structure, whereas the latter peak is attributed to the β-sheet conformation^9^,^10^,^11^,^12^,^13^,^14^,^15^,^16^,^17^,^18^,^19^,^20^,^21^,^22^,^23^,^24^,^25^,^26^,^27^. A peak at 1068 cm^−1^ is assigned to the C-C stretching vibration of the β-sheet conformation^26^. Additionally, multiple absorption peaks are also observed in the 2800–300 cm^−1^ region, corresponding to C-H stretching vibrations^28^,^29^,^30^. The observed peak at 1743 cm^−1^ corresponds to C=O stretching.

When 4HR is applied to silk, most of the amide peaks remain unchanged. However, several minor changes appear in the IR spectrum. The C-H vibrational absorption peaks are additionally strengthened due to the hydrocarbon chain of 4HR. An extra peak appears at 1026 cm^−1^ (indicated by the asterisk), which corresponds to the C-O vibration of 4HR^31^. One notable point is that the amide III peak corresponding to the β-sheet conformation is dramatically enhanced upon 4HR treatment, indicating an additional configuration of the β-sheet structure.

The 4HR-incorporated silk sutures showed a higher strain and a similar straight pull strength compared to the untreated silk sutures of the same size [Table 1]. However, both sutures had lower straight pull strengths compared to polyglactin 910. After 14 days of hydration, both silk sutures showed increased straight pull strength [Table 1]. The 4HR-incorporated silk sutures showed greater strain but lower knot-pull strength compared to untreated silk sutures of the same size [Supplementary Table S1]. Interestingly, all tested sutures showed lower knot-pull strength compared to straight pull strength. In the case of the knot-holding capacity, the 4HR-incorporated silk sutures showed the highest value among the three groups [Table 2].

### 4HR release profile from silk sutures and anti-microbial properties of 4HR-incorporated silk sutures

Supplementary Fig. S1 shows the release behavior of 4HR from the silk sutures in aqueous medium for an extended period of 7 days. The incorporated 4HR was rapidly released from the silk sutures during the initial 10 h, and more than 89% was released within 24 h, and reached a plateau after 48 h. We examined the volume effect of the extracted aqueous medium, but no obvious difference was observed in the measured time and volume scales. Considering the amount of incorporated 4HR (91.2 mg) in the silk sutures, the calculated released amount of 4HR (46.6 mg) means that approximately 50% of the 4HR was released from the silk sutures into solution after 7 days.

The silk disk with 4HR and the paper disk with 4HR showed an inhibition zone against all tested microbial species [Fig. 1b and Supplementary Table S2]. The 4HR-incorporated silk sutures also showed an inhibition zone against all tested microbial species [Fig. 1c and Supplementary Table S2]. By contrast, the bare silk disks and the silk sutures without 4HR did not show inhibition zones. The sizes of the inhibition zones in the 4HR-incorporated silk and in the paper disk with 4HR were smaller than that of tetracycline-loaded disks.

### *In vitro* proteolysis of silk fibroin and silk sutures by MMP-2, -3, and -9

**Figure 2:**
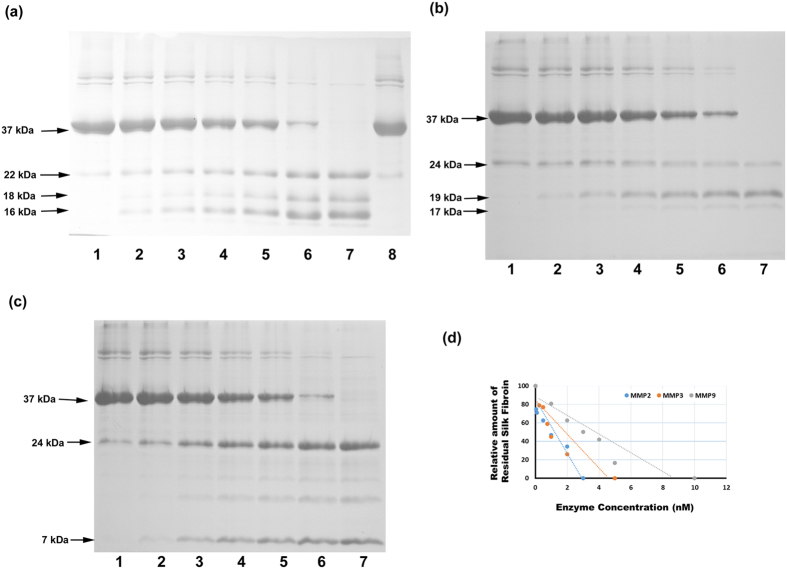
Proteolysis assay of silk fibroin. (**a**) Proteolysis of silk fibroin by MMP-2. MMP-2 degraded silk fibroin in a dose-dependent manner. Proteolysis of silk fibroin was completely blocked by an MMP-2 inhibitor (1: No enzyme, 2: 0.05 nM MMP-2, 3: 0.1 nM MMP-2, 4: 0.5 nM MMP-2, 5: 1 nM MMP-2, 6: 2 nM MMP-2, 7: 3 nM MMP-2, 8: 3 nM MMP-2 + 100 nM MMP-2 inhibitor). (**b**) Proteolysis of silk fibroin by MMP-3. MMP-3 degraded silk fibroin in a dose-dependent manner (1: No enzyme, 2: 0.25 nM of MMP-3, 3: 0.5 nM of MMP-3, 4: 0.75 nM of MMP-3, 5: 1 nM of MMP-3, 6: 2 nM of MMP-3, 7: 5 nM of MMP-3). (**c**) Proteolysis of silk fibroin by MMP-9. MMP-9 degraded silk fibroin in a dose-dependent manner (1: No enzyme, 2: 1 nM of MMP-3, 3: 2 nM of MMP-3, 4: 3 nM of MMP-3, 5: 4 nM of MMP-3, 6: 5 nM of MMP-3, 7: 10 nM of MMP-3). (**d**) The relationship between the applied enzyme concentration and the residual amount of protein. With increasing enzyme concentrations, the amount of residual protein decreased. The dotted line was drawn based on regression analysis.

Next, we tested MMP-2-mediated silk fibroin degradation. As shown in Fig. 2a, silk fibroin was degraded by MMP-2, and its proteolysis was inhibited by an MMP-2 inhibitor. MMP-3 and MMP-9 also degraded silk fibroin, but a slightly higher concentration of enzymes was required compared with MMP-2 [Fig. 2b,c]. The relationship between the applied enzyme concentration and the amount of residual protein amount was analyzed by linear regression [Fig. 2d]. The required amount of enzyme for complete proteolysis of 37 kDa sized silk fibroin protein within 2 h at 37 °C was calculated based on the results of the linear regression analysis [Supplementary Table S3]. The required amount of MMP-2, MMP-3, and MMP-9 were 3.03 nM, 4.41 nM, and 8.66 nM, respectively.

MMPs were administered to silk suture materials, and scanning electron microscopy (SEM) images were taken. The SEM images confirmed that MMP-2, MMP-3, and MMP-9 could induce the proteolysis of the silk suture materials [Supplementary Fig. S2]. The mechanical strengths were measured, and the 4HR-incorporated silk sutures had reduced mechanical strength after MMP treatment compared to bare silk sutures [Supplementary Table S4].

### MMPs-2, -3, and -9 induction in macrophages by 4HR and the bio-degradation of silk sutures

**Figure 3:**
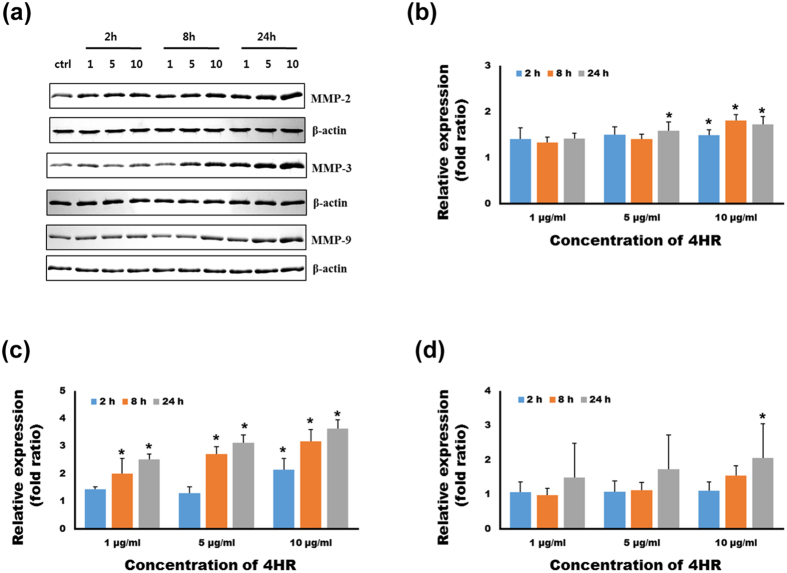
The results of the western blot. (**a**) 4HR increased the expression of MMP-2, -3, and -9 in RAW264.7 cells. The expression levels gradually increased for 24 h after 4HR administration. The relative expression levels of MMP-2 (**b**), -3 (**c**), and -9 (**d**) in 4HR-treated groups (*P < 0.05 compared to untreated controls).

**Figure 4:**
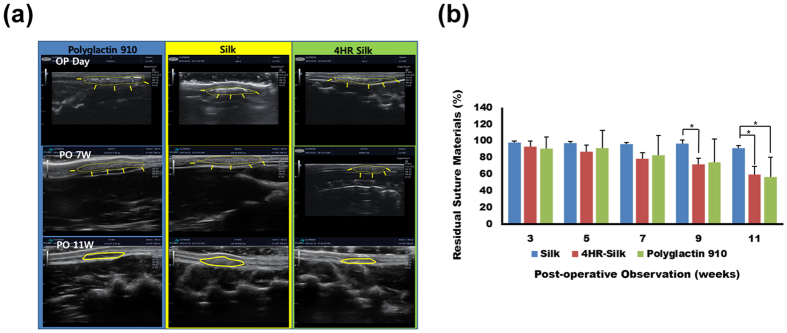
The results of the *in vivo* testing. (**a**) Serial images of ultrasonography. (**b**) The relative dimensions of the silk suture grafts were measured by ultrasonography. The dimension of the grafts at implantation was set to 100. Compared to the untreated silk group, the 4HR-silk group showed significantly smaller residual grafts at 9 and 11 weeks after surgery (^*^P < 0.05). For polyglactin 910, the residual graft was significantly smaller than the untreated silk group 11 weeks after surgery (^*^P < 0.05).

In this study, 4HR was administered to RAW264.7 cells, which is a cell line of murine macrophages, and a higher expression levels of MMP-2, -3, and -9 were observed compared to the untreated controls [Fig. 3]. Silk sutures, 4HR-treated silk sutures, and polyglactin 910 sutures were implanted under the skin of rats. Polyglactin 910, which is more commonly known as Vicryl^®^, is one of the most widely used bio-degradable suture materials. When assessed by ultra-sonography, both the 4HR-treated silk sutures and polyglactin 910 exhibited a gradual volume loss of grafts until 11 weeks after implantation [Fig. 4]. The results of immunohistochemical staining demonstrated that the expression of MMPs was not high in 4-week and 8-week samples for both the untreated silk and 4HR-treated silk groups [Supplementary Figs S3 and S4]. Interestingly, volume loss appeared to be faster starting 9 weeks after implantation according to sonography [Fig. 4]. The difference between the groups started to become significant after 9 weeks (P < 0.05).

**Figure 5:**
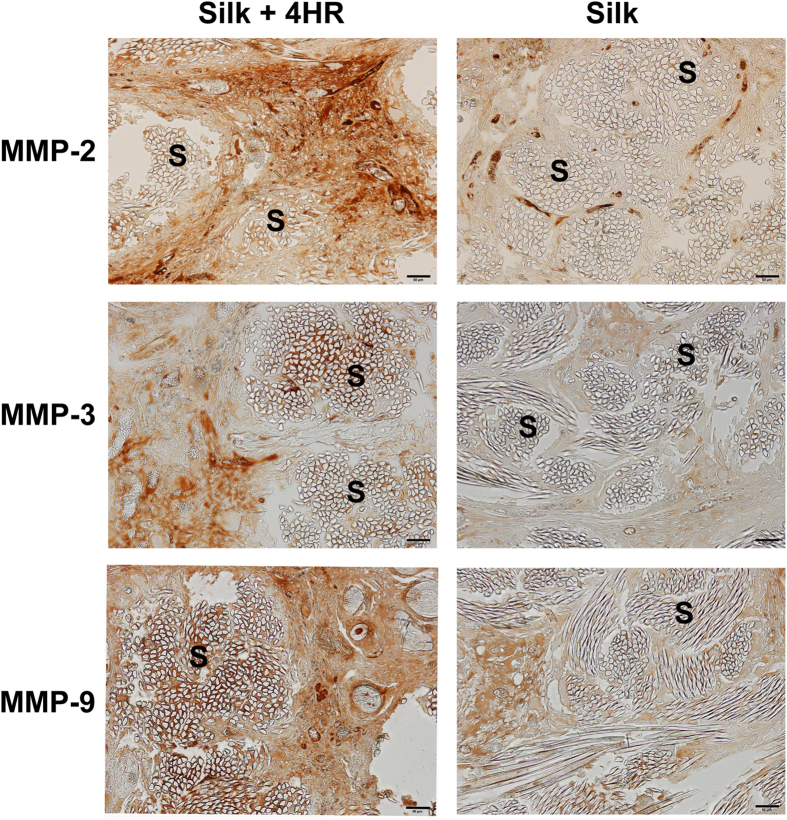
Immunohistochemical findings for tissue samples after 12 weeks. The expression levels of MMPs were higher in both groups compared to the groups at 4 and 8 weeks. MMPs were highly expressed in the fibrotic area that surrounded the silk sutures (S). Interestingly, highly expressed MMPs were observed in the vicinity of the silk sutures (S) in the silk +4HR group (Bar = 50 μm).

In a histological examination of 12-week samples, 5 of 9 samples of 4HR-treated silk suture samples showed almost complete degradation of the graft, and only a small amount of the materials was observed [Supplementary Fig. S5]. However, the silk suture group showed less degradation. In an immunohistochemical analysis, the suture material remaining in the samples from the 4HR-treated silk suture group showed higher expression levels of MMP-2, -3, and -9 than the silk suture group [Fig. 5]. Strong MMP expression was observed adjacent to the residual grafts. The untreated silk group also showed elevated expression levels of MMP-2, -3, and -9 compared to the 4-week and 8-week samples. However, the expression of MMPs was mainly observed in capsular fibrotic tissue. Upon comparing the relative intensity of immunostaining, the expression of MMPs in the 4HR-treated silk suture group was higher than that in the untreated silk suture group [Supplementary Fig. S6].

## Discussion

4HR has a hydrophobic long alkyl group and low solubility in water^11^. Its hydrophobic alkyl group can easily be incorporated into the hydrophobic domain of a biological molecule^32^. Because silk fibroin is mainly composed of hydrophobic domains^5^, 4HR can be easily incorporated into silk suture materials when the proper media are selected. In this study, 4HR was incorporated into commercially available silk suture materials, and its successful incorporation into silk sutures was examined based on FT-IR spectra. The FT-IR spectra of untreated silk sutures and 4HR-incorporated silk sutures are shown in Fig. 1a. For silk blended with 4HR, the relative absorption (600~1700 cm^−1^) of protein was enhanced compared to that of untreated silk. When silk sutures were treated with 4HR, the C-H (2,800–3,000 cm^−1^) and C-O (1,026 cm^−1^)^31^ vibrational absorption peaks, indicated by asterisks, were also strengthened due to the hydrocarbon chain of the 4HR, whereas most of the amide peaks remained. Extraordinarily, the amide III peak (1,261 cm^−1^) corresponding to the β-sheet conformation was significantly enhanced with 4HR treatment, which indicates that there were additional β-sheet structures [Fig. 1a].

The stability of the protein conformation may influence the physical strength of the sutures. The 4HR-incorporated silk sutures showed a higher strain and a similar yield strength compared to untreated silk sutures of the same size [Table 1]. Water molecules can bind to silk fibroin and change its conformation, which results in increased its physical strength^33^. In addition, salts such as K^+^ can induce the conformational change of silk fibroin from random coils to β-sheets^34^. Increased content of β-sheet in silk fibroin is related to improved mechanical properties^34^. Therefore, 4HR treatment did not weaken the tensile strength of the silk sutures. In addition, 4HR-incorporated silk sutures maintained their strength in normal saline at 37 °C for 2 weeks, which was similar to the behavior of unprocessed silk sutures [Table 1]. However, the tensile strength of polyglactin 910 sutures was decreased by approximately 20% after saline treatment [Table 1]. This result is because the polyglactin 910 sutures are degraded by hydrolysis^2^. The knot-holding capacity of 4HR-incorporated silk sutures was also higher than that of untreated silk sutures of the same size [Table 2].

Wound infection can be enhanced by foreign body reactions to suture materials^2^. Accordingly, anti-microbial sutures are beneficial in clinical practice, such as in cerebrospinal fluid shunt surgery^35^ and pediatric hand surgery^36^. Antibacterial sutures decrease healing time^37^. One-half of the 4HR loaded into the silk sutures was released within 48 h [Supplementary Fig. S1]. This burst release in the early period would be helpful for the suppression of bacterial growth [Supplementary Table S2]. 4HR-incorporated silk fibroin disks and 4HR-incorporated silk sutures showed anti-bacterial properties [Fig. 1b,c]. There have been several approaches used to develop anti-microbial sutures. Triclosan is an antimicrobial material and is used as a coating material for polyglactin 910 sutures^38^. Polyglactin 910 sutures with triclosan reduced implant infections to 25.8%^39^. The color of the 4HR-incorporated silk sutures changed to brown. Dye-like incorporation has been shown to prolong anti-microbial effects^40^. One-half of the 4HR incorporated into the silk sutures was released within 48 h, and the remaining was hardly released over the next 7 days. The release of hydrophobic drugs from silk fibroin is more sustained compared to hydrophilic drugs^41^. 4HR is an amphiphilic drug^11^, and silk fibroin has both hydrophobic block and hydrophilic parts^24^. Therefore, 4HR may adsorb to both the hydrophobic block and hydrophilic parts of silk sutures, and these interactions may explain the release pattern of 4HR from silk sutures.

4HR can enter the nuclei of cells^42^ and may influence gene expression. 4HR may also incorporates into the hydrophobic domain of proteins and affect protein function^32^. For lysozyme proteins, a low concentration of 4HR can increase enzyme activity through hydrophobic interactions^12^. Among the proteolytic enzymes, we hypothesized that the action of MMPs may be regulated by 4HR administration because 4HR modulates the nuclear factor-kappa B (NF-kB) signaling pathway^43^. Several MMPs are increased in macrophages via the NF-kB pathway^44^. MMPs can digest every type of extracellular matrix protein, such as collagen and elastin^45^. Because silk fibroin has a similar structure to the extracellular matrix protein, MMPs were selected as a candidate enzyme for silk fibroin degradation. MMP-2, -3, and -9 are proteolytic enzymes for collagen^46^. MMP-2 and MMP-9 have similar substrates, whereas MMP-3 has a wider spectrum of substrates than MMP-2 and MMP-9^46^. Macrophages are responsible for the phagocytosis of foreign materials. In this study, 4HR was administered to RAW264.7 cells, which is a cell line of murine macrophages, and higher expression levels of MMP-2, -3, and -9 were observed compared to untreated controls [Fig. 3]. Next, we tested MMP-2-mediated silk fibroin degradation. As shown in Fig. 2a, silk fibroin was degraded by MMP-2, and its proteolysis was inhibited by an MMP-2 inhibitor [Fig. 2]. MMP-3 and MMP-9 also degraded silk fibroin, but a slightly higher concentration of enzymes was required compared to MMP-2 [Fig. 2d].

The degradability of silk fibroin protein was different after the degumming process and subsequent manufacturing processes^19^. MMPs were administered, and SEM images were taken. The SEM images confirmed that MMP-2, MMP-3, and MMP-9 were able to induce proteolysis of the silk suture materials [Supplementary Fig. S2]. The mechanical strengths were measured, and 4HR-incorporated silk sutures were shown to have lower mechanical strength after the MMP treatment than bare silk sutures [Supplementary Table S4]. Natural polymer, such as silk protein, is absorbed during proteolysis^18^. Therefore, the mechanical strength of both silk suture groups was decreased after MMPs treatment [Supplementary Table S4]. Comparing the data in Table 1 to those in Supplementary Table S4, interestingly, the loss of mechanical strength was enhanced in the 4HR-incorporated silk sutures compared to bare silk sutures. In the case of MMP-2 treatment, 21.19% of ULTS was additionally lost in 4HR-incorporated silk suture compared to bare silk suture. Similar trends were also observed in the groups of MMP-3 and MMP-9 treatment. Additional loss of ULTS in the 4HR-incorporated silk sutures was 7.36% and 12.23% for MMP-3 and MMP-9, respectively. Enhanced degradation of the 4HR-incorporated silk sutures compared to untreated silk sutures demonstrated that 4HR could increase MMP activity. Lysozyme activity is increased depending upon 4HR concentration, which is due to protein conformational changes by the incorporation of 4HR into protein^12^.

Subsequently, animal experiments to test for the absorption of the suture materials were performed. Silk sutures, 4HR-treated silk sutures, and polyglactin 910 were implanted under the skin of rats. When assessed by ultra-sonography, both 4HR-treated silk sutures and polyglactin 910 showed gradual graft volume loss until 11 weeks after implantation [Fig. 4]. The results of the immunohistochemical staining demonstrated that the expression of MMPs was not high in the 4-week and 8-week samples for both the untreated silk and 4HR-treated silk groups [Supplementary Figs S3 and S4]. Interestingly, volume loss appeared to be faster starting at 9 weeks after implantation according to sonography [Fig. 4]. The difference between the groups first appeared to be significant after 9 weeks (P < 0.05). Proteolysis of the extracellular matrix is usually observed in the remodeling phase of skin wound healing, and the resolution of the inflammatory phase determines the transition to the next phase of wound healing^47^. The presence of poorly degrading grafts, such as those with silk sutures, may delay the transition of the phase from the inflammatory phase to the next phase. Silk sutures shows less change in its initial volume until 11 weeks after implantation^48^. The 4HR-treated silk sutures showed a similar level of bio-degradation compared to polyglactin 910 sutures [Fig. 4].

**Figure 6:**
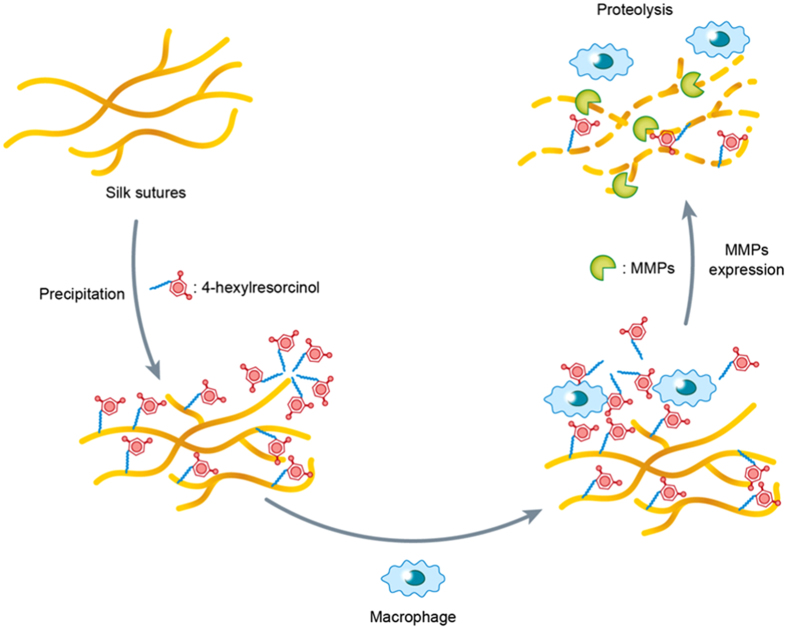
Proposed mechanism of suture degradation with the help of 4HR. Because 4HR is an amphiphilic chemical, it has both hydrophobic and hydrophilic regions. A hydrophobic long alkyl group can interact with the hydrophobic domain of silk sutures. To interact with the hydrophilic domain of silk sutures, 4HR competes with water, and a high-density 4HR micelle forms in vicinity of the hydrophilic domain of the silk suture through preferential hydration. Most of the high-density 4HR micelles are mostly released within a short period of time after implantation because the hydrogen bond of 4HR with silk protein is weak compared to that with water. Firmly bound 4HR in a hydrophobic domain is exposed during the wound remodeling phase by macrophages. The released 4HR induces MMPs in macrophages and produces MMPs that can digest silk suture material.

Collectively, 4HR increased the expression of MMP-2, -3, and -9 in macrophages. The increased MMP-2, -3, and -9 degraded silk fibroin and the silk sutures. The 4HR-treated silk sutures exhibited anti-microbial properties and a higher expression levels of MMP-2, -3, and -9 compared to untreated silk sutures *in vivo*. Thus, the MMP-mediated proteolysis of suture materials may have occurred more actively in the 4-HR-treated silk suture group than in the untreated silk suture group [Fig. 6]. Therefore, silk sutures incorporating 4HR represent a novel bio-degradable suture material with anti-microbial properties.

## Methods

### Silk sutures and 4HR incorporation

Silk suture material (4–0) was purchased from a commercial vendor (Woorhi Medical, Namyangju, Korea). Silk sutures were placed into a 3% 4HR solution. For maximum incorporation of 4HR into silk sutures, the mixture was placed in blended solvents on a rotating shaker for 1 h. Silk suture with 4HR was then dried in an oven at 60 °C for 4 h. The measured content of the 4HR in silk suture was approximately 12 wt%. Silk sutures with 4HR were sterilized with ethylene oxide gas and stored at room temperature until usage. A synthetic biodegradable suture material, polyglactin 910 (4–0, Johnson & Johnson, Diegem, Belgium) of a similar size was also purchased for comparison.

### FT-IR absorbance spectra

FT-IR spectra were measured using a FT-IR spectrometer (Vertex 80, Bruker Optics, KBSI, Daegu, Korea) equipped with an attenuated total reflectance (ATR) accessory (MIRacle, PIKE technologies, Madison, WI, USA). Vibrational absorption spectra were recorded in the spectral range of 800 to 4,000 cm^−1^ at a resolution of 4 cm^−1^ with 128 repeated scans using a DLaTGS detector.

### Knot-holding capacity and tensile strength

Sutures were tied between 2 hooks. The distance between the hooks was 10 cm. The location of the knot was positioned exactly between the two hooks of the tensiometer (Universal Testing Machine; RB302ML, R&B, Daejeon, Korea). The tension was applied to the suture at a rate of 10 mm/min. The tension was applied until loop breakage or slip of the knot. At that time, the knot holding capacity on the gauge and the knot status were recorded. This experiment was repeated 5 times with loops of each type of suture.

To measure straight pull tensile strength, each suture was cut to a length of 10 cm. Each end was fixed to the fixator of the tensiometer (Universal Testing Machine) at a length of 1 cm. The gauge length was set at 8 cm. The tension speed was 10 mm/min (n = 5). The same experiments were performed to measure the knot-pull tensile strength.

### 4HR release behavior from silk sutures

The 4HR release behavior was studied based on ultraviolet-visible (UV-Vis) absorption spectroscopy using a spectrophotometer (Varian Cary 5 G, Agilent, Santa Clara, CA, USA)^49^. The loading capacity of 4HR-incorporated silk sutures was 12 wt%. The sutures were immersed in 1 L of phosphate-buffered saline (PBS, pH = 7.4, 25 °C), and then the released amount of 4HR was estimated as a function of time, by observing the absorbance increment at 280 nm and using the absorption coefficient of 4545 cm^−1^ M^−1^ of 4HR in the release medium [Supplementary Fig. S1a]. In addition, 91.2 mg 4HR-incorporated silk suture material was also placed in fresh 200 ml of PBS (pH = 7.4, 25 °C) to inspect the solvent volume effect on the release of 4HR. The released 4HR solution was diluted by 20 times prior to absorption measurements to avoid spectrum saturation. For each measurement, all the release medium was replaced with fresh medium.

### Anti-bacterial test

Three pathogens (*Staphylococcus aureus, Streptococcus sanguinis,* and *Actinomyces naeslundii*) were used for antibacterial tests of drug-loaded disks. The subsequent procedure was in accordance with a previous publication^14^. The silk disks, 4HR-incorporated silk disks (12 wt%), and 4HR-incorporated paper disks were placed on the surface of brain heart infusion (Becton, Dickinson and Company, Sparks, MD, USA) agar plate. For comparison, an antibiotic disk (tetracycline) was also used. Six pathogens (*S. aureus, S. sanguinis, A. naeslundii, Streptococcus gordnonii, Escherichia coli*, and *Actinomyces odontolyticus*) were used for the anti-bacterial tests of drug-loaded silk sutures. Silk suture knots and 4HR (12 wt%)-incorporated silk suture knots were placed on the culture plate.

Images of each culture plate were taken using a ChemiDoc XRS system (Bio-Rad Laboratories, Hercules, CA, USA). To enhance contrast, pseudocolor was applied to the images, and the size of the inhibition zone was measured as the maximum diameter.

### MMPs and silver staining

RAW264.7 cells (Korean Cell Line Bank No. 40071) are murine macrophages. RAW264.7 cells were suspended in culture medium. The detailed procedure for the western blot analysis was the same as reported in our previous publication^16^. RAW264.7 cells were placed in six-well culture plates and 1, 5, and 10 μg/ml of 4HR was applied. After 2, 8, and 24 h of culture, the cells were collected. The same volume of solvent without 4HR was applied to the control culture. The collected proteins were mixed with a sodium dodecyl sulfate buffer and were denatured by heating. They were electrophoresed in 10% polyacrylamide gels. The gels were transferred to a polyvinylidene difluoride membrane. After blocking the membranes, the blots were probed with a primary antibody (dilution ratio = 1:500). The sources and specifications of primary antibodies were as follows: MMP-2 (Abcam, Cambridge, U.K.), MMP-3 (Abcam), MMP-9 (Abcam), and β-actin (Sigma-Aldrich, St. Louis, MO, USA). The images were taken and quantified using a ChemiDoc XRS system (Bio-Rad Laboratories). A representative blot was collected, and the relative mean intensity ± standard deviation of three independent blots normalized to β-actin were used for analysis. The untreated control was set to 1. The relative expression levels in each experimental group were statistically compared to the untreated control group.

Silk fibroin digestion was carried out as described in a previous publication^50^. The silk fibroin protein was prepared by the Rural Development Administration. MMP-2 and MMP-3 were purchased from PeproTech (Rocky Hill, NJ, USA), and MMP-9 was purchased from R&D systems (Minneapolis, MN, USA). Silk fibroin (300 ng) was incubated with varying amounts of MMP-2, -3, or -9 as indicated in 10 μl of buffer containing 20 mM HEPES, pH 7.4, 140 mM NaCl, and 2 mM CaCl_2_ for 2 h at 37 °C. The samples were then soaked by adding 10 μl of 20 mM EDTA. ARP100 (Santa Cruz Biotech, Santa Cruz, CA, USA) was used as an MMP-2 inhibitor. The samples were run on 5% SDS–PAGE gels followed by silver staining. For the quantitative analysis of the proteolysis mediated by MMPs, the most intense band observed in untreated control was selected and its relative intensity after enzyme treatment was measured by Sigma Scan Pro^®^ (Chicago, IL, USA).

Proteolysis of the silk sutures was performed as described in a previous publication^19^. Briefly, MMP-2, -3, and -9 were reconstituted to 100 μg/ml in 50 mM HEPES, 10 mM CaCl_2_, and 0.05% Brij-35 buffer, and the pH was adjusted to 7.5. For SEM examination, 20 mm of silk or 4HR-incorporated silk suture material was placed into 10 μl of reconstituted enzyme and 1 ml of Hank’s balanced salt solution in a 37 °C water bath for 3 days. For physical strength examination, 0.3 g silk or 4HR-incorporated silk suture material was placed into 10 μl of reconstituted enzyme and 10 ml of Hank’s balanced salt solution in a 37 °C water bath for 3 days. The tensile strength was then measured as described above.

### Animals and surgical procedures

#### Degradation analysis of 4HR-treated silk sutures in rats

Forty-five 12-week-old Sprague-Dawley rats with an average weight of 300 mg were used for this experiment. A 2-cm longitudinal incision was made in the back skin. Silk suture material or 4HR-incorporated silk suture material was grafted into the subcutaneous pocket area. In each group, 5 rats were examined at 4 weeks, 5 rats at 8 weeks, and 10 rats at 12 weeks. Polyglactin 910 suture material was grafted into the subcutaneous pocket area, and the rats were sacrificed at 12 weeks (n = 5). The weight of the graft was set as 0.03 g. All animal procedures were carried out in accordance with the guidelines issued by the Institutional Animal Care and Use Committee (GWNU-2015-6).

#### Doppler sonography

On the day of operation day and at 3, 5, 7, 9, and 11 weeks post-operation, we used an ultrasonic machine (ACCUVIX V10^®^, Samsung Medison, Seoul, Korea) to evaluate the residual graft in the subcutaneous pocket. First, the spinal bone was identified. The fatty layer was then identified over the spinal bone. The graft was localized between the connective tissue and the fatty layer. An image of the residual graft was recorded and used for image analysis. The ratio of the residual graft was calculated as the percent ratio between the graft size on post-operative day 1 and the graft size of the observation.

#### Histomorphometric evaluation

Skin samples were embedded in paraffin blocks. The paraffin blocks were sliced into sections that were then stained with hematoxylin and eosin. For immunohistochemical staining of MMPs, the slides were de-waxed and incubated with a selected antibody at 4 °C overnight. After DAB staining, a cover slip was placed without counterstaining. Quantitative analysis of immunohistochemistry was performed in accordance with our previous publication^51^.

#### Statistical analysis

SPSS for Windows ver. 19 (IBM Co., Armonk, NY, USA) was used for the statistical analysis. The differences between the mean values of each group were evaluated by independent sample t-tests. Linear regression analysis was used to evaluate proteolysis by MMPs. The level of significance was set at P < 0.05.

### Ethical approval and informed consent

The animal experiments were approved by the Institutional Animal Care and Use Committee of Gangneung-Wonju National University (GWNU-2015-6).

## Additional Information

**How to cite this article**: Jo, Y.-Y. *et al*. Accelerated biodegradation of silk sutures through matrix metalloproteinase activation by incorporating 4-hexylresorcinol. *Sci. Rep.* **7**, 42441; doi: 10.1038/srep42441 (2017).

**Publisher's note:** Springer Nature remains neutral with regard to jurisdictional claims in published maps and institutional affiliations.

## Supplementary Material

Supplementary Information

## Figures and Tables

**Table 1:** Straight pull strength of sutures before and after 14 days of normal saline treatment.

	Polyglactin 910		Silk		4HR-Silk	
	Before	After	Before	After	Before	After
ULTS (MPa)	817.17 ± 20.87	576.82 ± 22.56	338.85 ± 13.82	407.26 ± 5.23	314.79 ± 11.17	383.10 ± 24.85
Tensile strain (%)	29.90 ± 1.69	24.12 ± 1.18	13.82 ± 2.25	20.79 ± 0.41	21.33 ± 1.24	24.04 ± 1.91

(ULTS: ultimate longitudinal tensile strength).

**Table 2:** Knot-holding capacity before and after 14 days of normal saline treatment.

	Polyglactin 910		Silk		4HR-Silk	
	Before	After	Before	After	Before	After
Knot holding capacity (N)	2.20 ± 0.59	5.86 ± 2.11	4.06 ± 1.32	5.44 ± 1.58	6.85 ± 0.43	6.43 ± 0.96
