# Supplementary material for: Accelerated biodegradation of silk sutures through matrix metalloproteinase activation by incorporating 4-hexylresorcinol
Source: Sci Rep. 2017 Feb 13;7:42441. doi: 10.1038/srep42441 (PMC5304327; doi:10.1038/srep42441)
Supplement: Supplementary Information [file srep42441-s1.pdf]

Accelerated bio-degradation of silk suture through matrix metalloproteinase activation by incorporating 4-hexylresorcinol

You-Young Jo[1], HaeYong Kweon[1], Dae-Won Kim[2], Min-Keun Kim[3], Seong-Gon Kim[3, *], Jwa-Young Kim[4], Weon-Sik Chae[5], Sam-Pyo Hong[6], Young-Hwan Park[7], Si Young Lee[8], and Je-Yong Choi[9]

[1]Rural Development Administration

[2]Dept. of Oral Biochemistry, College of Dentistry, Gangneung-Wonju National University

[3]Dept. of Oral and Maxillofacial Surgery, College of Dentistry, Gangneung-Wonju National University

[4]Dept. of Oral and Maxillofacial Surgery, College of Medicine, Hallym University

[5]Analysis Research Division, Daegu Center, Korea Basic Science Institute, South Korea

[6]Dept. of Oral Pathology, College of Dentistry, Seoul National University

[7]Department of Biosystems & Biomaterials Science and Engineering, Seoul National University, South Korea.

[8]Dept. of Oral Microbiology, College of Dentistry, Gangneung-Wonju National University

[9]School of Biochemistry and Cell Biology, Skeletal Diseases Genome Research Center, Kyungpook National University, Daegu 700-842, Korea

*Corresponding authors
E-mail address: kimsg@gwnu.ac.kr, Fax: +82-33-641-2477

Supplementary Figures

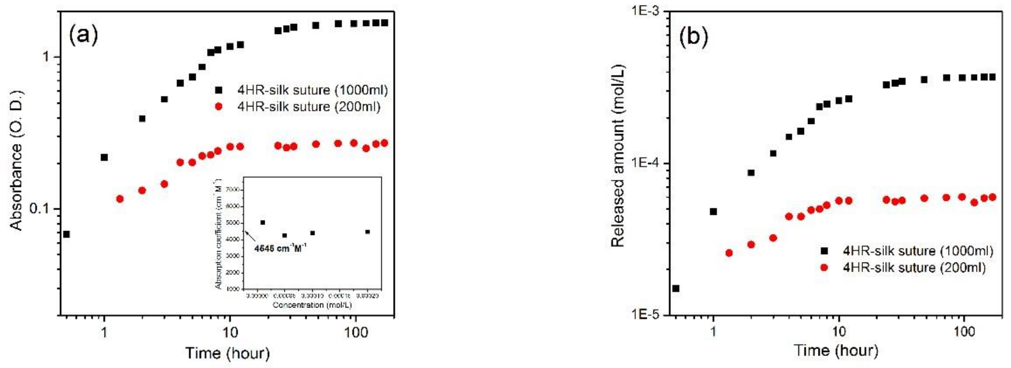

Supplementary Figure S1. Drug release pattern. (a) Absorbance and (b) released amount of 4HR from silk suture as a function of time. The released 4HR in 200 ml water was diluted by 20 times prior to absorption measurements to avoid spectrum saturation. In this case, the released 4HR amount was calculated to be 46.6 mg in 200 ml water until 7 days.

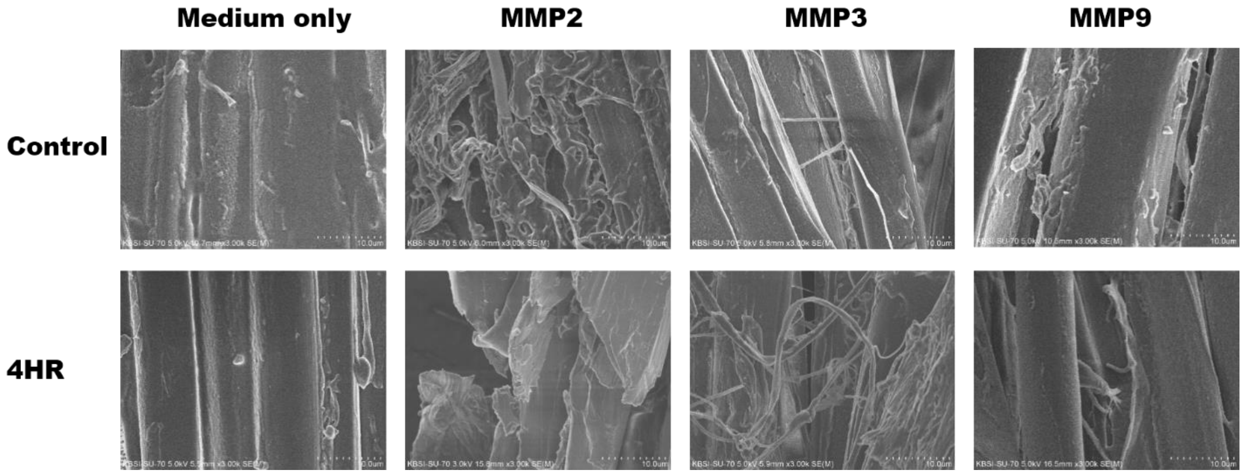

Supplementary Figure S2. MMPs were administered, and scanning electron microscopy (SEM) images were taken. The SEM images also demonstrated that MMP-2, MMP-3, and MMP-9 could achieve a proteolysis of the silk suture materials. The degree of proteolysis appeared to be severe in the silk + 4HR group compared to the untreated silk group.

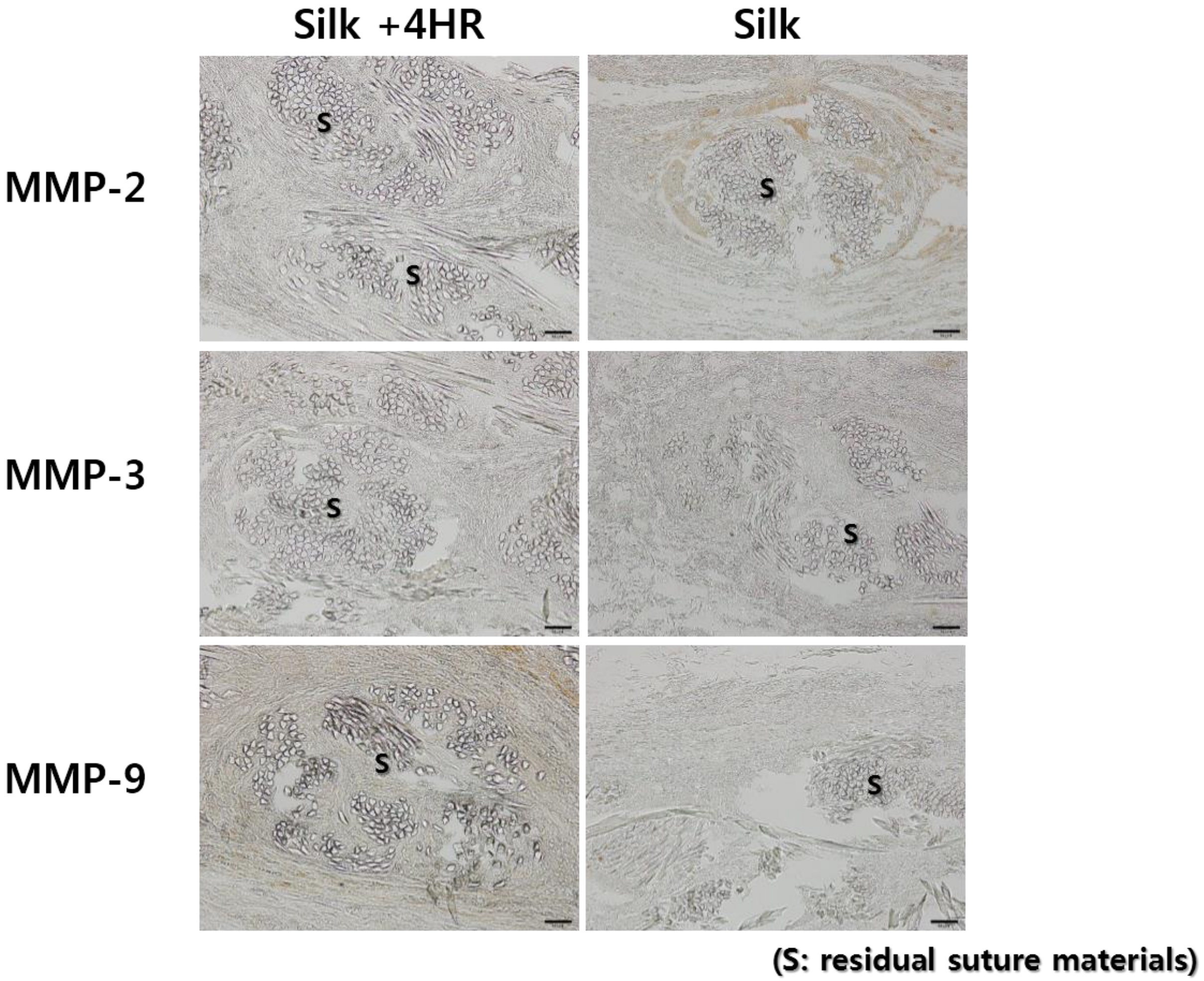

Supplementary Figure S3. Immunohistochemical findings for tissue samples at 4 weeks. The expression levels of MMPs were very low in both groups (Bar=50 μm).

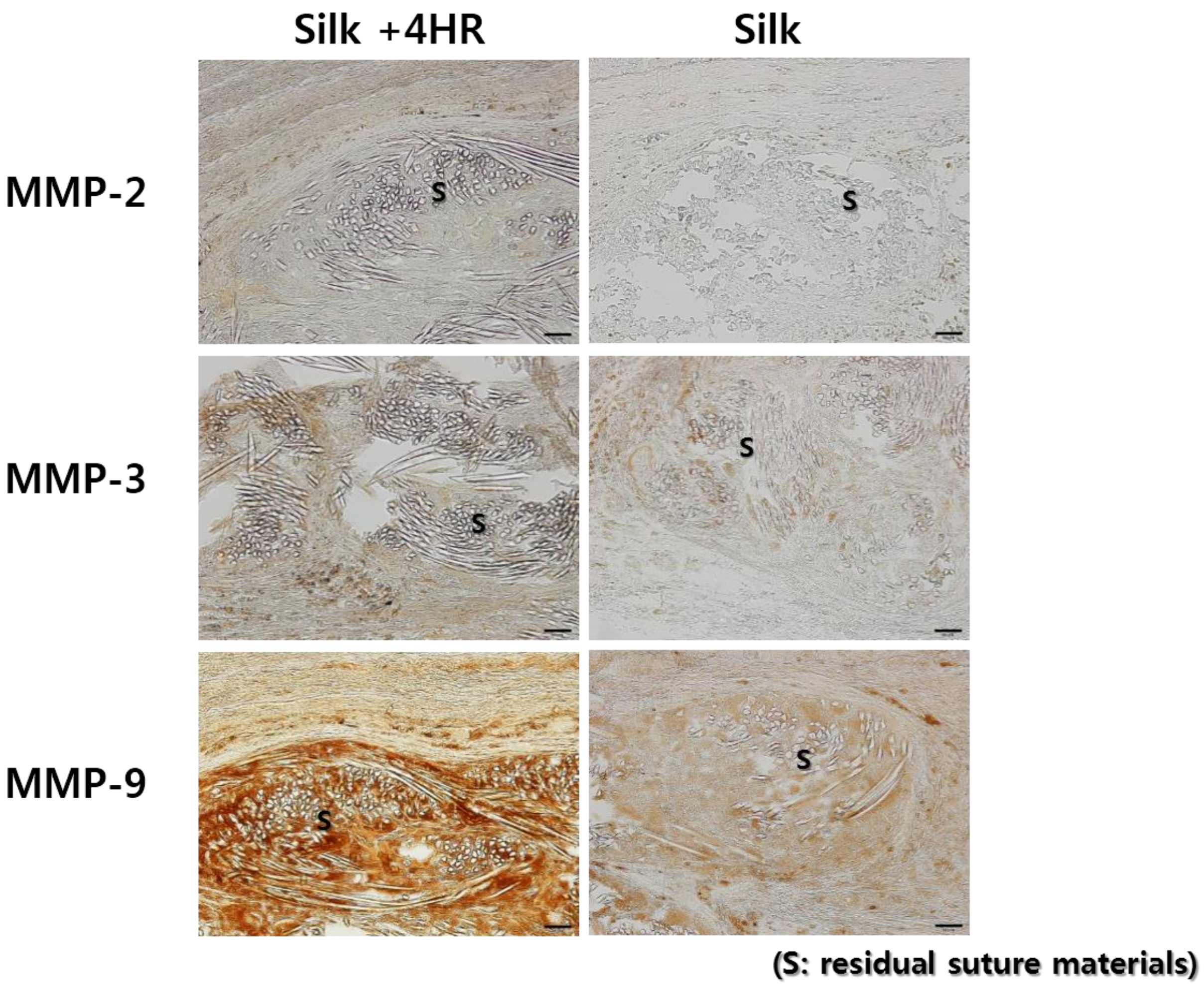

Supplementary Figure S4. Immunohistochemical findings for tissue samples after 8 weeks. The expression levels of MMP-2 and MMP-3 were low. The expression level of MMP-9 was relatively high compared with MMP-2 and -3 (Bar=50 μm).

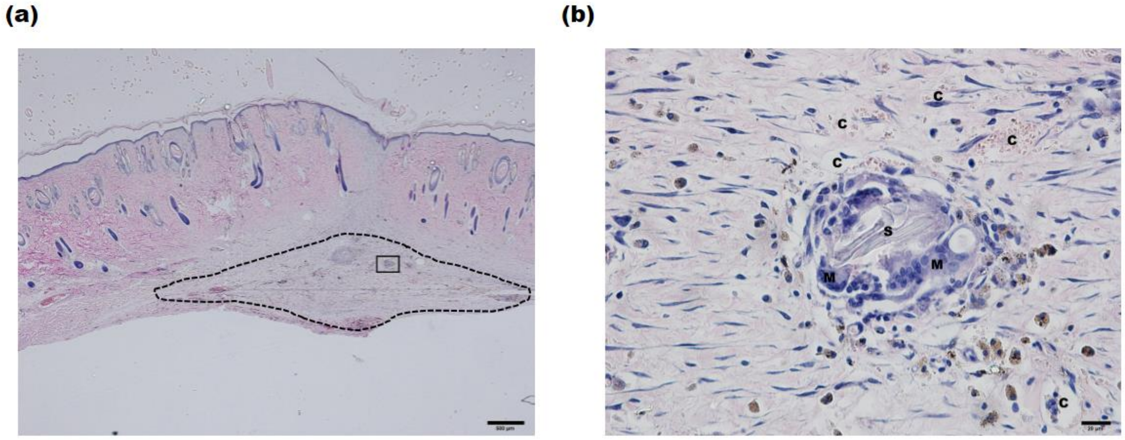

Supplementary Figure S5. Histological view of 4HR-treated silk suture at 12-weeks after operation (hematoxylin and eosin staining). (a) The graft was almost degraded. Remnants of suture materials were sporadically observed in the grafted area (dotted line, bar= 500 μm). (b) High magnification view of rectangular area of (a). Residual suture material (S) was surrounded by macrophages or multi-nucleated cells (M). Many capillaries (C) were also found. It might be due to elevated level of MMPs (bar= 20 μm).

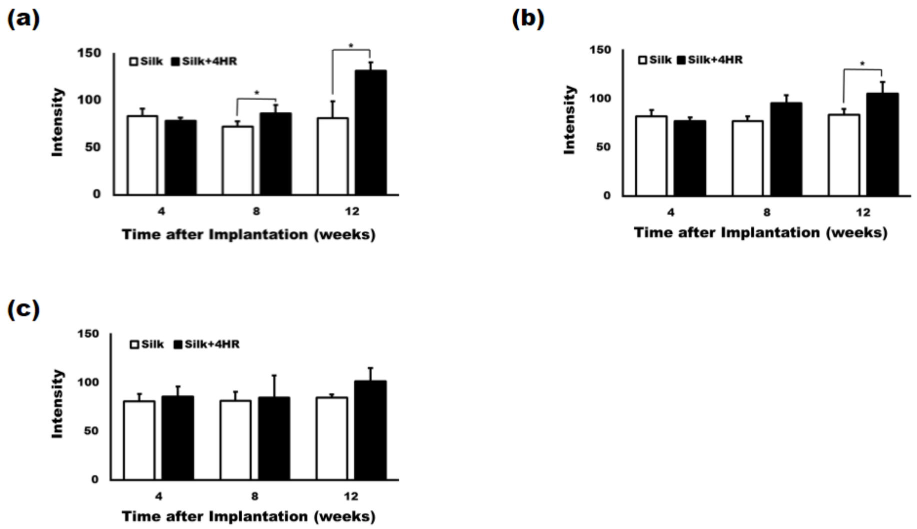

Supplementary Figure S6. Quantitative analysis of immunohistochemical staining. (a) The intensity of immuno-staining for MMP-2. Compared to bare silk sutures, specimens from the 4HR-incorporated silk suture group showed significantly higher staining intensity at 8 weeks and 12 weeks after grafting (*$P<0.05$). (b) The intensity of MMP-3 immuno-staining. Compared to bare silk suture, specimens from the 4HR-incorporated silk suture group showed significantly higher staining intensity at 12 weeks after grafting (*$P<0.05$). (c) The intensity of MMP-9 immuno-staining. Compared to bare silk suture, specimens from the 4HR-incorporated silk suture group showed higher staining intensity at 12 weeks after grafting. However, the difference between groups was not significantly different ($P=0.05$).

Supplementary Tables

Supplementary Table S1. Knot-pull strength of sutures

	Polyglactin 910	Silk	4HR-Silk
ULTS (MPa)	292.50 ± 32.40	265.80 ± 40.24	187.82 ± 14.75
Tensile strain (%)	19.58 ± 0.81	13.81 ± 1.97	14.93 ± 0.69

(ULTS: ultimate longitudinal tensile strength)

Supplementary Table S2. Bacterial inhibition zone

Species	TC	Silk disk	Silk + 4HR disk	4HR disk	Silk suture	Silk + 4HR suture
S. aureus	41 mm	0	32 mm	31 mm	0	20 mm
S. sanguinis	37 mm	0	30 mm	25 mm	0	14 mm
A. naeslundii	50 mm	0	40 mm	38 mm	0	26 mm
A. odontolyticus	34 mm	Not tested	Not tested	Not tested	0	20 mm
S. gordonii	22 mm	Not tested	Not tested	Not tested	0	15 mm
E. coli	12 mm	Not tested	Not tested	Not tested	0	3 mm

(TC: tetracycline)

Supplementary Table S3. Estimates of the linear regression between proteolytic enzyme and silk fibroin degradation

Enzyme		B	SD	t	Sig.	95% CI	
						Lower	Upper
MMP-2	Slope	-26.76	3.77	-7.11	0.001	-36.44	-17.08
	Constant	81.13	5.37	15.10	<0.001	67.31	94.94
MMP-3	Slope	-18.04	3.68	-4.90	0.004	-27.51	-8.58
	Constant	79.63	7.73	10.30	<0.001	59.76	99.51
MMP-9	Slope	-9.90	1.61	-6.15	0.002	-14.04	-5.76
	Constant	85.70	7.58	11.31	<0.001	66.22	105.19

(SD: standard deviation, Sig: significance, CI: confidence interval)

Supplementary Table S4. Straight pull strength of sutures after MMP treatment

	MMP-2		MMP-3		MMP-9	
	Silk	Silk+4HR	Silk	Silk+4HR	Silk	Silk+4HR
ULTS (MPa)	192.02 ± 37.05	111.70 ± 22.24	196.84 ± 27.60	159.69 ± 12.59	212.68 ± 32.70	159.08 ± 31.96
Tensile strain (%)	16.03 ± 6.38	14.14 ± 4.13	13.66 ± 3.14	13.03 ± 2.15	12.69 ± 2.99	11.34 ± 3.86

(ULTS: ultimate longitudinal tensile strength)
